# Impacts of Gaseous Ozone (O_3_) on Germination, Mycelial Growth, and Aflatoxin B_1_ Production In Vitro and In Situ Contamination of Stored Pistachio Nuts

**DOI:** 10.3390/toxins14060416

**Published:** 2022-06-17

**Authors:** Alaa Baazeem, Angel Medina, Naresh Magan

**Affiliations:** 1 Department of Biology, College of Science, Taif University, P.O. Box 11099, Taif 21944, Saudi Arabia; aabaazeem@tu.edu.sa; 2 Applied Mycology Group, Environment and AgriFood Theme, Cranfield University, Cranfield MK43 0AL, UK; a.medinavaya@cranfield.ac.uk

**Keywords:** gaseous ozone, germination, growth, aflatoxin B_1_, water availability, *Aspergillus flavus*, pistachios

## Abstract

Pistachio nuts can become colonized by mycotoxigenic fungi, especially *Aspergillus flavus*, resulting in contamination with aflatoxins (AFs). *We* examined the effect of gaseous O_3_ (50–200 ppm; 30 min; 6 L/min) on (a) in vitro germination, (b) mycelial growth, and (c) aflatoxin B1 (AFB_1_) production on a milled pistachio nut-based medium at different water activity (a_w_) levels and at 30 °C. This was complimented with in situ studies exposing raw pistachio nuts to 50–200 ppm of O_3_. Exposure of conidia to gaseous O_3_ initially resulted in lower germination percentages at different a_w_ levels. However, 12 h after treatment, conidial viability recovered with 100% germination after 24–48 h. Growth rates of mycelial colonies were slightly decreased with the increase of the O_3_ dose, with significant inhibition only at 0.98 a_w_. The production of AFB_1_ after O_3_ treatment and storage for 10 days was stimulated in *A. flavus* colonies at 0.98 a_w_. Raw pistachio nuts inoculated with *A. flavus* conidia prior to O_3_ exposure showed a significant decrease in population after 20 days of storage. However, AFB_1_ contamination was stimulated in most O_3_ treatments. The relationship between exposure concentration, time and prevailing a_w_ levels on toxin control needs to be better understood for these nuts.

## 1. Introduction

Ozone (O_3_) is commonly generated in the summer because of the reactions between photochemicals in the atmosphere in the presence of sunlight. O_3_ gas has been commonly used in the food industry for sanitizing packaging materials, raw materials, and storage facilities [1]. It has been suggested that the advantage of O_3_ over other remedial chemicals used in foodstuffs is that it is residue-free. It decomposes to diatomic oxygen rapidly because of its short half-life, which is about 20–50 min in the atmosphere and 1–10 min in water [2]. However, it is a very corrosive gas, and for its use, it is critical that appropriate tubing (PTFE), steel, or glass systems are used. Manning and Teidemann [3] showed that small increases in ozone (O_3_) concentrations (40–60 ppb) can influence the mycobiota of plant surfaces and perhaps the biosynthesis of toxins. Slight increases in exposure to gaseous O_3_ as a pollutant was previously shown to change the phyllosphere mycobiota on conifer needles [4]. 

At the present time, the EU legislation established a maximum level of 12 μg/kg aflatoxin B_1_ (AFB_1_) in nuts, including pistachios used for processing, and 8 μg/kg AFB_1_ for direct human consumption [5]. The AF contamination of pistachio nuts occurs either pre-harvest, because of early splitting of the shells and insect damage, or during subsequent drying and processing [6]. For example, in Iran, an analysis of 10,000+ samples of pistachio nuts showed AFB_1_ in 36%, with almost 12% exceeding the maximum level in Iran (5 μg/kg) [7]. In Algeria, between 1998 and 2002, the highest AFB_1_ in 523 samples was 113 μg/kg, with a mean range of 1 to 3.8 μg/kg [8]. In Tunisia, 76% of pistachio nuts were found to be contaminated with AFs during storage [9]. Delayed harvesting and processing and storage has been shown to significantly influence and often increase the AFB_1_ contamination of pistachio nuts [6,10]. Thus, there has been interest in examining treatments such as gaseous O_3_ for the mitigation of AFs in nuts, including pistachios [11,12,13].

Some previous studies have examined the efficiency of electrochemically-generated O_3_ on the activity of aflatoxigenic fungi and aflatoxin (AFs) in different nuts, including peanuts and brazil nuts. However, there are less studies on pistachio nuts [11,12,13]. Mylona et al. [14] showed that gaseous O_3_ (100–400 ppm) was not very effective against the microconidal germination of *Fusarium verticiilioides* in vitro, but it was able to reduce fumonisin B_1_ (FB_1_) contamination in stored maize grain after treatment. Indeed, the initial inhibition of germination was followed by recovery, growth, and FB_1_ production. However, Sultan et al. [12] found that gaseous O_3_ was very effective in inhibiting the germination of conidia of *A. flavus*, but it had little efficacy in controlling the mycelial growth of *A. flavus* strains on peanut-based media, regardless of water activity (a_w_). Studies have been carried out on the O_3_ treatment of Brazil nuts and showed that exposure to this gas affected the growth of the mycobiota and decreased AFs [11]. They used three concentrations of O_3_ (10, 14, and 31.5 mg/L) for five hours and found that this inhibited colonization by both *A. flavus* and *A. parasiticus* over a storage period of 180 days. However, at low concentrations of O_3_, the fungi were still able to grow. Care is needed with nuts because the high concentration of lipids can interact with gaseous O_3_, resulting in tainting and off-flavors [15]. The sensitivity of fungal species to O_3_ exposure may vary and depends on the exposure period, concentration applied, and type of commodity. It can also be influenced by moisture content and spore morphology [16,17].

No previous studies have examined the effect of gaseous O_3_ (0–200 ppm) as a control measure for germination, colonization, and AFB_1_ in raw milled pistachio-based media or in stored raw pistachios. Thus, the aim of this study was to examine the use of gaseous O_3_ to control (a) in vitro conidial germination, (b) in vitro mycelial growth and AFB_1_ production, and (c) *A. flavus* populations on raw pistachio nuts when inoculated with conidia of this species and exposed to O_3_ and the effects on subsequent AFB_1_ contamination after 20 days of storage at different a_w_ levels.

## 2. Results

### 2.1. Effect of O_3_ on Conidial Germination of A. flavus In Vitro on a Milled Pistachio-Based Medium

**Figure 1:**
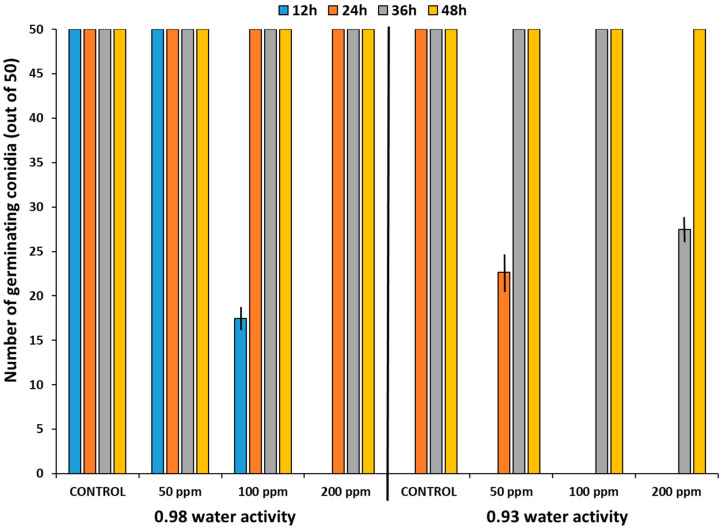
Effect of gaseous ozone (6 L/min for 30 min) on the mean number of conidia of *A. flavus* strain AB3 germinating (mean of 9 × 50 conidia) at 0.98 and 0.93 a_w_ on a 3% milled raw pistachio nut medium and then incubated at 30 °C. Bars indicate the SEM number of spores germinated.

The mean germination of conidia of *A. flavus* was influenced by gaseous O_3_ treatment, regardless of the a_w_ level used (Figure 1). However, all the O_3_-treated conidia showed a recovery in germination capacity after 48 h, regardless of a_w_ level. The only exceptions were the samples treated with 200 ppm O_3_ at 0.98 a_w_ and all O_3_ doses at 0.93 a_w_ after 12 h. 

### 2.2. In Vitro Effect of Gaseous O_3_ Treatment on Mycelial Growth

**Figure 2:**
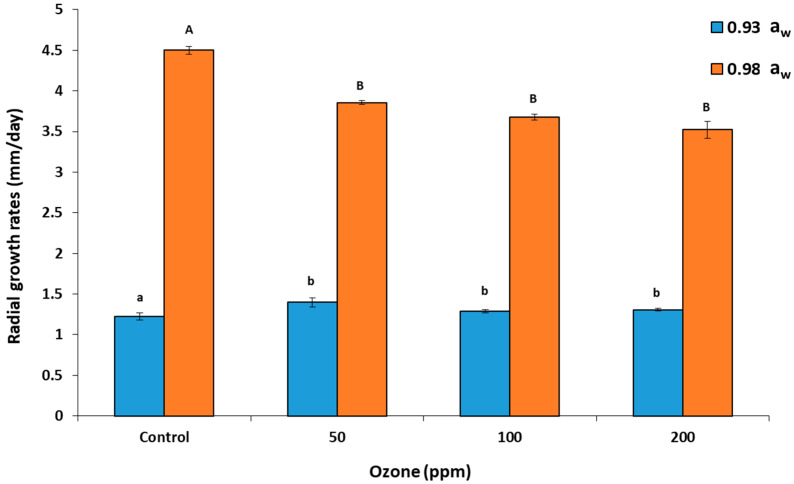
Effect of gaseous ozone treatment on the mycelial colony extension rate of *A. flavus* (strain AB3) on a 3% milled raw pistachio nut-based medium at 0.98 and 0.93 water activity (a_w_) and 30 °C for 4–6 days. Colonies were exposed to gaseous ozone for 30 min at 6 L/min prior to incubation. Different letters indicate significant differences (*p* < 0.05). Bars indicate mean SE.

The mycelial extension of *A. flavus* (strain AB3) was significantly inhibited after O_3_ treatment when compared with the control at 0.98 a_w_ (Figure 2). However, the growth rates with the different O_3_ exposure concentrations were relatively similar. At 0.93 a_w_, growth rates were slightly higher after O_3_ treatment, with little difference between the concentrations of O_3_ used.

### 2.3. In Vitro Effect of Gaseous O_3_ Exposure of Colonies of A. flavus on AFB_1_ Production

**Figure 3:**
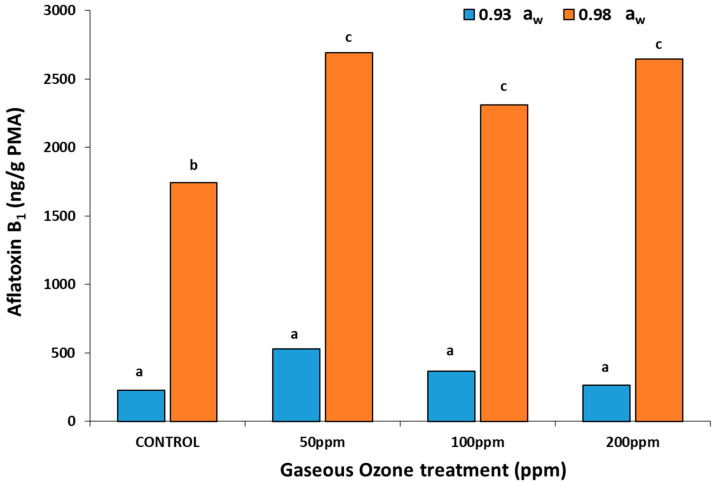
Effect of ozone treatment on AFB_1_ production by *A. flavus* (strain AB3) at 0.93 and 0.98 a_w_ on a raw milled pistachio-based medium after 10 days at 30 °C. Colonies were exposed to ozone for 30 min at 6 L/min prior to incubation. Different letters indicate significant differences (*p* < 0.05).

The AFB_1_ productions were significantly higher after O_3_ treatments at all exposure levels when compared to the untreated control in the 0.98 a_w_ treatments (Figure 3). However, at 0.93 a_w_, there was no significant effect on toxin production, with much lower AFB_1_ productions on the raw pistachio-based medium. 

### 2.4. In Situ Effects of Gaseous O_3_ Treatment on Populations of A. flavus in Stored Raw Pistachio Nuts

**Figure 4:**
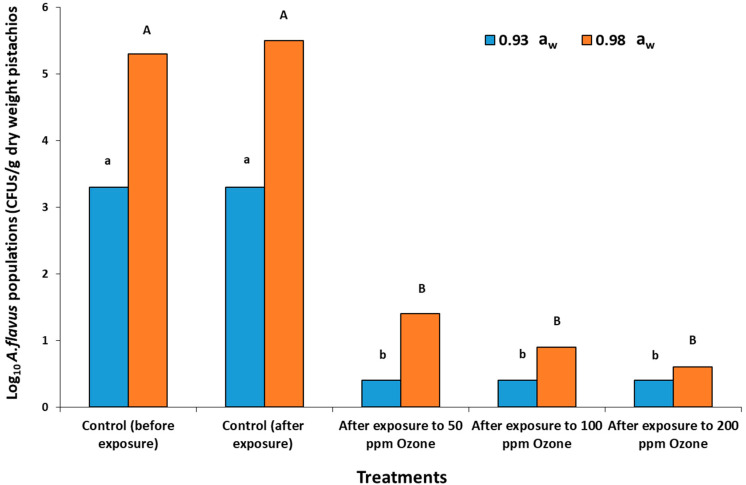
Populations of *A. flavus* (strain AB3, Log_10_ CFUs) isolated from ozonized raw pistachio nut samples before and after treatment and storage for 20 days. Exposure to O_3_ was for 30 min at 6 L/min prior to incubation. Different letters indicate significant differences (*p* < 0.05).

The total populations of *A. flavus* slightly increased in the control samples when exposed to air (Figure 4). However, there was a significant reduction in the *A. flavus* CFUs from Log_10_ 5.3 CFUs (control after exposure to air) to Log_10_ 1.4 CFUs, after exposure to 50 ppm O_3_, and <Log_10_ 1.0 CFUs after 100 and 200 ppm O_3_ exposure at 0.98 a_w_. For pistachio nuts at 0.93 a_w_, the *A. flavus* populations remained very similar regardless of the gaseous O3 treatment. At both the a_w_ levels, it seemed that higher O_3_ doses (100, 200 ppm) did not have any increased efficacy when compared to 50 ppm O_3_ exposure. 

### 2.5. In Situ Effects of O_3_ Treatment on AFB_1_ Contamination of Raw Stored Pistachio Nuts

**Figure 5:**
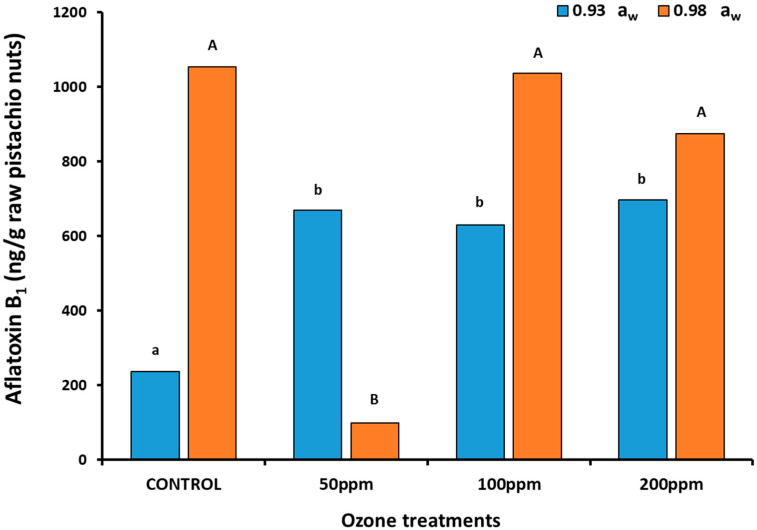
Effect of ozone exposure on AFB_1_ contamination of raw pistachio nuts inoculated with *A. flavus* strain AB3 at 0.93 and 0.98 a_w_ then stored for 4 weeks after treatment at 30 °C. The pistachio nuts were exposed to gaseous ozone for 30 min at 6 L/min prior to storage. Different letters indicate significant differences between treatments (*p* < 0.05).

The AFB_1_ contamination was found to always be higher in the treated samples compared to the controls at both a_w_ levels and all gaseous O_3_ exposure levels, except for 50 ppm (Figure 5). At this concentration and 0.98 a_w_, the AFB_1_ was significantly reduced. 

## 3. Discussion

### 3.1. In Vitro Effects on Germination and Mycelial Extension

The efficacy of gaseous O_3_ for the control of conidial germination and mycelial growth of *A. flavus* strains (AB3, AB10) was evaluated in this study on a milled raw pistachio nut-based medium at different a_w_ levels. The a_w_ levels chosen were based on ecological data, which showed that optimum *A. flavus* growth occurs at >0.97 a_w_ and optimum AFB_1_ at >0.94 a_w_ at 25–30 °C. For the prevention of toxin contamination, pistachio nuts would need to be dried to <0.88 a_w_ [18]. Overall, exposure of conidia to O_3_ initially had lower germination percentages when compared to the controls at both the a_w_ levels. Treatment with 200 ppm O_3_ at 0.98 a_w_ showed the complete inhibition of germinations after 12 h; however, spore viability appeared to recover, and the germination was increased after 24 h and reached 100% germination after 48 h. Sultan and Magan [12] found that there was an effective inhibition of conidial germination by O_3_ treatment of *A. flavus* strains from peanuts with complete inhibition at 200–250 ppm O_3_ (6 L/min; 30 min). However, this was on a defined yeast extract sucrose medium. Previously, Mylona et al. [14] examined the in vitro effect of O_3_ treatment at 100 and 200 ppm for 30 min (6 L/min) on the spore germination of *F. verticillioides*. Although germinative capacity was inhibited after 24 h, over the following 72 h there was a recovery, and after 8–10 days at both 0.98 and 0.94 a_w_, FB_1_ production occurred. They also found that doubling the exposure time (60 min) did not improve the efficacy of O_3_. Indeed, recent studies with species from the *Aspergillus* section *Circumdati* and *Nigri* responsible for the ochratoxin A contamination of coffee showed tolerances of up to 500 ppm O_3_ [19]. It has been suggested that O_3_ acts by oxidizing vital cellular components, especially unsaturated lipids in cell membranes, resulting in a leakage of cell contents and subsequent microbial lysis at high concentrations [20,21]. However, some of these studies have been performed in O_3_-treated water and not with gaseous O_3_ [12].

For mycelial growth, the present study showed that the mycelial extension was inhibited by O_3_ exposure at 0.98 a_w_. However, growth rates decreased only slightly with the increasing O_3_ dose. Very few studies have examined the in vitro effect on the growth of *A. flavus*, and none were on nut-based media [12]. A previous study by Zotti et al. [22] found that O_3_ treatment of 3-day-old *A. flavus* colonies for 3 h inhibited growth and spores completely. However, when the same colony reached 6 and 9 days old, the efficacy decreased. Additionally, they found that there are different sensitivity levels among species, with *A. flavus* being less sensitive than *A. niger*. However, in their study, a_w_ modification was not considered and the O_3_ concentration used was only 1 ppm. Akbar et al. [19] found that the mycelial extension of strains of *A. carbonarius* and *A. westerdijkiae* in coffee-based medium with up to 500 ppm of O_3_ had little effect on the rates of colonization, regardless of a_w_ level used or exposure period (30–60 min). 

The general tolerance of aflatoxigenic and ochratoxigenic *Aspergillus* spp. to O_3_ may in part be due to the darker pigmentation and relatively thick-walled conidia, which can provide protection against UV-light, sunlight, and toxic gases. In addition, the capacity for relatively rapid DNA repair after exposure may be quite rapid, allowing the viability of conidia to be conserved after exposure. This could be related to both pigmentation and/or repair systems that help the cells to recover viability. Indeed, Hibben and Stotzky [16] indicated that small hyaline spores are more sensitive to O_3_, while large and pigmented spores, such as the conidia of *A. niger*, were more resistant [23,24]. Spores of *A. fumigatus* have been found to be particularly resistant to O_3_ [18]. In contrast, spores of *Fusarium* species (e.g., *F. verticillioides*, *F. langsethiae*), which are practically hyaline and have very little pigmentation, appear to be sensitive to O_3_ exposure in air initially, although some recovery of viability was found [9]. A thorough comparison amongst species belonging to the same genus is important. 

Reduction of fungal growth can be obtained in high moisture conditions after treatment with up to 1000–15,000 ppm O_3_ for 1 h [25]. For Brazil nuts, O_3_ treatment was found to affect the growth of the mycobiota and to decrease aflatoxin contamination levels [26]. However, the exposure period was 5 h, which was effective and inhibited the growth of *A. flavus* and *A. parasiticus*, although they were still able to grow during the initial few days after O_3_ exposure. Thus, O_3_ levels and exposure time together with the other influencing factors, including temperature and ERH, need to be examined in more detail to optimize the potential use of this gas for control of the key life cycle phases of mycotoxigenic spoilage fungi and toxin production [21].

### 3.2. In Vitro and In Situ Effects of O_3_ on AFB_1_ Contamination 

In the present study, AFB_1_ was analyzed after the in vitro exposure of colonies of *A. flavus* to O_3_ for 30 min and then stored for 10 days at 30 °C. There appeared to be variable effects on AFB_1_ production by exposure to O_3_ treatment. The increase in toxins may be due to O_3_ exposure acting as an environmental stress, resulting in the biosynthesis of more toxins as a defense reaction. In addition, it may be that the O_3_ interacts with the pistachio-based medium, changing the nutritional make up, especially in relation to fatty acids. Sultan and Magan [12] examined *A. flavus* exposure to O_3_ (100–300 ppm) on a conducive YES medium. In this case, the use of a defined medium and exposure to O_3_ resulted in a significant decrease of AFB_1_ production in mycelial colonies. However, they examined toxin biosynthesis after 3 days, while the present study examined it for 10 days on a pistachio-nut based medium to simulate the natural nutritional conditions as far as was possible. This could explain the differences observed. Previously, Mason et al. [2] showed that the exposure of *A. flavus* colonies for 5 days inhibited asexual conidial sporulation. This suggested that perhaps the effect of O_3_ on the whole life cycle of *A. flavus* would provide useful information on which phase might be more sensitive to such treatment [27]. The present study is the first to examine in detail the in situ effect of gaseous O_3_ on the colonization and toxin production by *A. flavus* in stored pistachio nuts. Overall, while to populations of *A. flavus* significantly decreased due to O_3_ exposure, there was little difference between 50–200 ppm treatment levels. Indeed, a reduction in AFB_1_ was only observed in the 50 ppm O_3_ × 0.98 a_w_ treatment. It may be that a reduction in overall populations of *A. flavus* allows more rapid subsequent colonization by the surviving inoculum of the rich nutrient source. In naturally contaminated pistachio nuts, the mycobiota is varied with a range of fungi present [28]. Thus, the niche will be occupied by a fungal community including *A. flavus*, and it would have to compete with these other fungi, some of which may survive O_3_ treatment. A previous study exposed pistachio nuts and ground nuts artificially contaminated with aflatoxins to very low concentrations of O_3_ (5–9 mg/L = 2–5 ppm gaseous O_3_) for 140 and 420 min [10]. They found that AFB_1_ and total aflatoxins were reduced by about 23 and 24%, respectively, with the highest O_3_ treatment level (9 mg/L) for 420 min. The treatments were much less effective against ground pistachios [15]. However, these studies were only carried out at 70% relative humidity, and spiking with the toxins is not the same as natural occurrence in this commodity due to colonization by the mycotoxigenic species. Thus, further studies are required to better understand the relationship between commodity type, exposure concentration × time of exposure, and prevailing a_w_ level to determine efficacy in terms of toxin control [13]. 

The studies by Mylona et al. [14] certainly suggest that the natural contamination of maize grain with fumonisins can be reduced by exposure to O_3_ concentrations. However, it may be more difficult to reduce mycotoxin production by specific fungi without longer term exposure to O_3_. Indeed, even with 500 ppm, O_3_ was found to have relatively little effect on the reduction of ochratoxin A contamination of stored green coffee contaminated with *A. westerdijkiae* and *A. carbonarius* and stored under different a_w_ levels at 30 °C [19]. Savi et al. [27] found that the exposure of wheat to 40–60 mg/L of O_3_ for up to 180 min reduced the growth of *F. graminearum* significantly, with deoxynivalenol in the pericarp and endosperm tissue being completely inhibited. However, the moisture content of the grain was not considered in these studies, which are important relative to colonization by *Fusaria* and trichothecene production. It is, however, important to consider that food ozonation may not always be a beneficial process, especially where this gaseous treatment may alter food sensory characteristics, color, cause lipid oxidation, and the degradation of phenolic compounds and vitamins [29,30]. 

## 4. Conclusions

This study has shown that the use of gaseous O_3_ exposure of both conidia and mycelial colonies of *A. flavus* on a milled raw pistachio nut-based medium was not very effective. Indeed, via repair systems, the conidia recovered germinative capacity rapidly. Up to 200 ppm of gaseous O_3_ for 30 min had little impact on mycelial extension and subsequent AFB_1_ production. In situ studies with stored raw pistachio nuts inoculated with *A. flavus* conidia and exposed to O_3_ reduced the isolation of the *A. flavus* populations but had little effect on AFB_1_ contamination after 20 days of storage at different a_w_ levels. This suggests that perhaps much longer exposure times may be required and that the efficacy may also be influenced by the moisture content and the type of commodity, in this case a lipid-rich matrix. However, this would have to be balanced against potential tainting effects and eating quality, which might be impacted if high exposure levels are used for longer time periods. In the future, for more effective and safe use in food processing, the optimum gaseous O_3_ concentration, contact time, and other treatment conditions need to be defined for specific foods. Pilot scale tests would probably need to be conducted for each commodity before potential commercial application, as every food application with O_3_ application may be different. 

In addition, in vitro and in vivo toxicological tests need to be conducted to quantify the effects of degradation products on human and animal health.

## 5. Materials and Methods

### 5.1. Apparatus for Ozone Generation and Experimental System

O_3_ was generated in the laboratory using a C-Lasky series O_3_ generator purchased from AirTree Ozone Technology Co. (model CL010DS), Sijhih, Taiwan. This equipment generates O_3_ by corona discharge between two tubs, with no metals involved for efficiency improvement, generation stability, and less energy consumption. The generated O_3_ was directed into the exposure chamber using a Teflon tube, which was properly connected to the generator. For safety reasons, the experiment was carried out in a fume cupboard to prevent O_3_ from spreading into the laboratory atmosphere. Two different systems were used for O_3_ exposure for in vitro and in situ assays. O_3_ concentration was measured using an O_3_ analyzer (Model UV-100, Eco Sensor, Santa Fe, NM, USA), which was connected to the chamber to measure the exit gas accurately. It should be noted that 1 ppm of O_3_ generated is equivalent to 2.14 mg/L of O_3_ in the air. This allows for comparison with some other studies. Experimental set-ups were performed as follows:

(a) The exposure system of O_3_ for in vitro germination and mycelial growth assays was a 5-L airtight glass jar. The O_3_ inlet of the system was connected from the generator to the lid of the jar using a Teflon tube, which was inserted into the bottom of the jar. The outlet of the system was also in the lid of the jar and connected to the O_3_ analyzer using a Teflon tube. This ensured accurate measurement of the O_3_ concentrations in the glass container. The flow rate of the generated O_3_ used was 6 L/min for 30 min.
(b) Exposure system of O_3_ for the in situ study.

The exposure chamber for in situ experiments was a 100-mL volume glass tube. The tube was capable of containing about 45–50 g of pistachio nuts. These were placed inside the column, and the O_3_ was forced upwards via an inlet at the bottom of the tube coming from the generator. The outlet at the top was connected to the O_3_ analyzer and vented in the fume hood. This allowed accurate exposure of the pistachio nuts to the treatment O_3_ concentrations for the necessary residence time. The flow rate of generated O_3_ was 6 L/min for 30 min.

### 5.2. Fungal Strains, Media, Spore Suspension, and Water Activity

One strain of *A. flavus* (AB3), representative of 3–4 others isolated from pistachio nuts, was used in these studies [13,31]. This was chosen as it was representative of those studied previously [13,31]. Pistachio-based media were used for spore germination and mycelial growth studies. A 3%-milled raw pistachio nut agar (PMA) was used with 2% technical agar (Thermo Fisher Scientific Oxoid Ltd., Basingstoke, Hampshire, UK) [13]. 

For spore suspension, fresh cultures of the AB3 strain were prepared on PMA and incubated at 25 °C for 5–7 days. AB3 culture surfaces were gently scraped and transferred into sterile Universal vials containing sterile water + 0.1% Tween 80 solution (Tween 80 (ACROS organics). The concentration of the spore suspension was determined using a hemocytometer (Olympus BX40 microscope, Microoptical Co., Sauerlach, Germany; slide Marienfeld superior, Germany; microscope glass cover slips, No 3, 18 mm × 18 mm, Chance proper Ltd., Malvern, Worcestshire, UK) and adjusted by dilution to 10^7^ spores/mL. Target a_w_ values (0.98 and 0.93) for PNA were obtained by using glycerol/water solutions, instead of water, to modify the a_w_ with this non-ionic solution. For these two treatments, the equivalent of 122.5 and 355 g of glycerol per L of water was used. This was mixed, and the mixture was used similar to water.

### 5.3. Effect of O_3_ on Conidial Spore Germination of A. flavus

Four different treatments were examined, including three concentrations of O_3_: 0, 50, 100, 200 ppm of O_3_ at the two different a_w_ levels detailed previously and incubated at 30 °C. Samples exposed to the air were used as controls for each a_w_. The experiment was carried out in triplicate and repeated once. An amount of 100 µL of 10^6^ spore suspension was spread onto PNA media treatments and replicates and allowed to dry. Lids were taken off the plates and the media were placed inside the airtight glass jar for O_3_ exposure for 30 min at a flow rate of 6 L/min, as described previously. The Petri plates were separated by 2–3 cm to ensure exposure of each plate. After exposure, plates were placed into plastic boxes, which were maintained at the same a_w_ levels with glycerol/water solutions (500 mL × 2) and stored at 30 °C. Three agar plugs were taken every 12 h from each plate using a surface-sterilized cork-borer (1 cm) and placed on a glass microscope slide (Fisherbrand, Leicestershire, UK). The agar plugs were then stained with Lactophenol Cotton Blue (ProLab Diagnostics, Birkenhead, UK) and covered with a glass coverslip. Each plug was then examined under the microscope, and germination was recorded. Spores were considered to have germinated when the length of the germ tube was longer than the diameter of the spore. A total of 3 × 50 single spores per replicate were examined (450 per treatment). The overall mean number of germinated spores (out of 50) was calculated for the different O_3_ treatments. 

### 5.4. In Vitro Effects of Gaseous O_3_ on Mycelial Growth and AFB_1_ Contamination

PNA media were inoculated centrally with 10 µL of spore suspension made from AB3 strain and incubated at 30 °C in replicates and allowed to grow for 2 and 5–6 days in the 0.98 and 0.93 a_w_ treatments, respectively. Measurements of colonies were recorded, and plates were exposed to O_3_ with no lids for 30 min using the system described previously. The O_3_ concentrations were 50, 100, and 200, with air as a control. A_w_ of media and ERH during incubation after O_3_ exposure was adjusted to 0.93 and 0.98 a_w_. After exposure, Petri plates were covered with the lids and incubated at 30 °C. Colony diameters were recorded on a daily basis for each treatment and compared with the control. Agar plugs were taken after day ten from each replicate and stored at −20 °C for AFB_1_ analysis. 

### 5.5. In Situ Effect of Gaseous O_3_ on Fungal Population and AFB_1_ Production on Irradiated Pistachio Nuts

Irradiated raw pistachio nuts (12–15 KGys; Synergy Health Sterilisation UK Ltd., Swindon, Wiltshire, UK) were weighed and place in sterilized bottles (eight bottles) for each treatment (40 g per replicate). The absence of any fungal contaminants was checked by direct-plating individual pistachio nuts on Malt Extract Agar medium (MEA; Thermo Fisher Scientific Oxoid Ltd., Basingstoke, Hampshire, UK) and incubated for 7 days at 25 °C. This showed no contamination. The raw pistachio nuts were rewetted using a moisture adsorption curve [25] and mixed well and left overnight at 4 °C to equilibrate to the target a_w_ levels of 0.93 and 0.98. A conidial suspension of 10^6^ spores was prepared. After equilibration, 1 mL of the spore suspension was added to the pistachio nuts and mixed well. Small sub-samples were taken (1 g) and placed in 10 mL of sterile water containing tween 80 in a 25 mL Universal bottle for serial dilution to assess the populations of *A. flavus* present. Three separate replicates of each treatment (40 g each) were exposed to O_3_ (50, 100, and 200 ppm, or air) for 30 min at a flow rate of 6 L/min. 

Immediately after exposure, sub-samples were taken for serial dilution of the populations. The rest of the pistachio nuts were placed in solid culture vessels with microporous lids (Magenta, Sigma Ltd., Coventry, UK). These were previously autoclaved at 121 °C for 15 min with aluminum foil covers. The glass chambers containing the treatments/replicates were then placed in plastic chambers with glycerol/water solutions to maintain the target ERH (93 and 98% ERH) and stored for 20 days at 30 °C. Samples were taken for *A. flavus* fungal populations after this storage period. The remaining pistachio nuts were stored at −20 °C for later AFB_1_ analysis. 

For serial dilution, samples were soaked for 20 min and then vigorously shaken using a vortex mixer. From each treatment/replicate serial dilutions were made. For each concentration, three replicates were made, and 100 µL was spread on MEA media using a sterile spreader and incubated at 30 °C for 7 days before colonies were counted for *A. flavus* populations. 

### 5.6. Aflatoxin B_1_ Quantification 

Preparation of aflatoxin standards: A 200-μL stock solution of aflatoxins (B_1_, B_2_, G_1_, G_2_) standard in methanol containing 250 ng AFB_1_ was prepared and pipetted into 2-mL Eppendorf tubes for overnight evaporation until dryness in a fume hood similar to the samples.

#### 5.6.1. In Vitro Aflatoxin B_1_ Analyses

Colony Extraction: Initially, agar plugs were cut out across the diameter of colonies using a surface-sterilized 4-mm diameter cork-borer (approx. 4–6). The agar plugs were placed in pre-weighed 2-mL Eppendorf tubes and weighed again. Five-hundred μL of HPLC-grade chloroform was added to the tubes and shaken for 30 min using a KS 501 digital orbital shaker (IKA (R) Werke GmbH & Co. KG, Esslingen, Germany). The chloroform extract was transferred to a new Eppendorf tube and dried gently under air for derivatization.

Derivatization of aflatoxin B_1_ extract: Derivatization of the AFB_1_ extract was performed according to the AAOC method [32]. First, 200 μL of hexane was added to the tube, followed by 50 μL of trifluoroacetic acid. The mixture was vortexed for 30 s and left for 5 min. A mixture of water:acetonitrile (9:1) was then added to the tube, vortexed for 30 s, and left for 10 min to allow for separation of the layers. Then, the aqueous layer was filtered using a syringe nylon filter (13 mm × 0.22 μm; Jaytee Biosciences Ltd., Herne Bay, UK) into amber-salinized 2-mL HPLC vials (Agilent, Santa Clara, CA, USA) before HPLC analysis. All analytical reagents used were HPLC-grade.

Quantification of aflatoxin B_1_ with High Performance Liquid Chromatography HPLC: A reverse-phase HPLC with fluorescence detection was used to confirm the identity and quantify AFB_1_. An Agilent 1200 series HPLC system was used for the analysis. It consisted of an in-line degasser, auto sampler, binary pump, and a fluorescence detector (excitation and emission wavelengths of 360 and 440 nm, respectively). Separation was achieved using a C18 column (Phenomenex Gemini; 150 × 4.6, 3 μm particle size; Phenomenex, Torrance, CA, USA) with a Phenomenex Gemini C18 3 mm, 3-μm guard cartridge. Isocratic elution with methanol:water:acetonitrile (30:60:10, *v*/*v*/*v*) as the mobile phase was performed at a flow rate of 1.0 mL/min. The injection volume was 20 µL. A set of standards was injected (1 to 5 ng AFB_1_, AFB_2_, AFG_1_, and AFG_2_ per injection), and standard curves were generated by plotting the area underneath the peaks against the amounts of AFB_1_ standard injected. The run time for the each HPLC analysis was 12 min. Supplementary Figure S1 shows an example of a standard and a sample for aflatoxin B_1_ quantification. 

#### 5.6.2. Quantification of Aflatoxin B_1_ in Pistachio Nuts

The pistachio nut samples were all dried in a drying oven at 50 °C in the dark. They were subsequently ground (Waring blender, Merck Ltd., Feltham, UK) and weighed (25 g). The background aflatoxin B_1_ level in the nuts used in the experiments was 0.015 ng/g. This was taken into account as a correction factor in the final quantification of the results. Acetonitrile/water 60/40 (100 mL) was used as an extraction solvent. The mixture was blended for 3 min, and the extract was filtered into a smaller sample container. PBS buffer (pH 7.4, Thermo Fisher Scientific) was used for sample dilution, then the diluted extract was passed through an Immunoaffinity Column (IAC; AflaStar™; Romer Labs, Tulln an der Donau, Austria) with a flow rate between 1–3 mL/min. The column was rinsed with 2 × 10 mL sterile distilled water. HPLC-grade methanol (1.5–3 mL) was then applied to the column, and the eluent was collected in a new amber glass vial and left to dry overnight at room temperature before the derivatization step, as detailed previously. 

### 5.7. Statistical Analysis

Three replicates per treatment were used in all studies and repeated once. Means were obtained by taking the average of three measurements for each experiment with the standard error of the means (±SE; standard error) obtained. Analysis of variance (ANOVA) was applied to analyze the variation of means with a 95% confidence interval. Normal distribution of data was checked by the normality test Kolmogorov-Smirnov, using Minitab statistical software. Fisher’s Least Significant Difference (LSD) was used to identify differences between the means, with *p* < 0.05 as a significant difference, using the same statistical software.

## Data Availability

The raw data used in this study and the statistical analyses are available via the corresponding author at Cranfield University.

## Supplementary Materials

The following supporting information can be downloaded at: https://www.mdpi.com/article/10.3390/toxins14060416/s1, Figure S1: Examples of HPLC analyses of (a) a standard and (b) a sample of pistachio nut-based agar for aflatoxin B_1_ quantification.
