# Supplementary material for: Impacts of Gaseous Ozone (O3) on Germination, Mycelial Growth, and Aflatoxin B1 Production In Vitro and In Situ Contamination of Stored Pistachio Nuts
Source: Toxins (Basel). 2022 Jun 17;14(6):416. doi: 10.3390/toxins14060416 (PMC9227268; doi:10.3390/toxins14060416)
Supplement: Supplementary file 1 [file toxins-14-00416-s001.zip › toxins-1759521-supplementary.pdf]

toxins

Impacts of Gaseous Ozone (O_3) on Germination, Mycelial Growth, and Aflatoxin B_1 Production In Vitro and In Situ Contamination of Stored Pistachio Nuts

Alaa Baazeem, Angel Medina and Naresh Magan

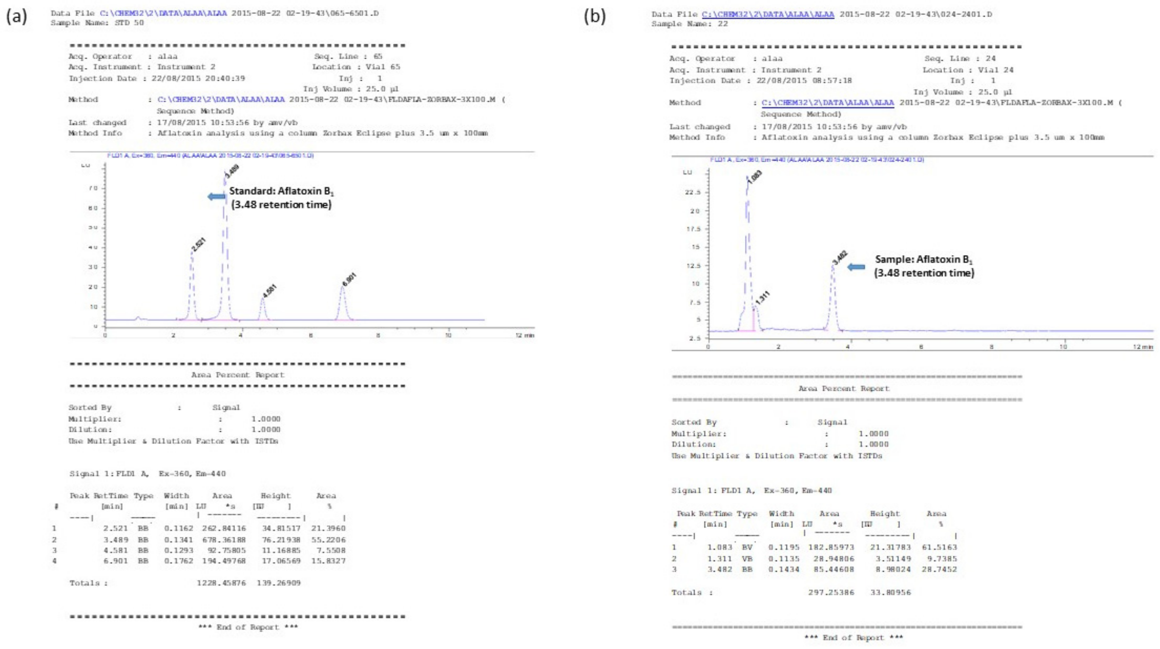

Figure S1. Examples of HPLC analyses of (**a**) a standard and (**b**) a sample of pistachio nut-based agar for aflatoxin B_1 quantification.
